# New Insights from 22-kHz Ultrasonic Vocalizations to Characterize Fear Responses: Relationship with Respiration and Brain Oscillatory Dynamics

**DOI:** 10.1523/ENEURO.0065-19.2019

**Published:** 2019-05-07

**Authors:** Maryne Dupin, Samuel Garcia, Julie Boulanger-Bertolus, Nathalie Buonviso, Anne-Marie Mouly

**Affiliations:** 1 Lyon Neuroscience Research Center, Institut National de la Santé et de la Recherche Médicale Unité 1028, Centre National de la Recherche Scientifique Unité Mixte de Recherche 5292, University Lyon 1, Lyon 69366, France

**Keywords:** fear response, oscillations, piriform cortex, prefrontal cortex, respiration, ultrasonic vocalizations

## Abstract

Fear behavior depends on interactions between the medial prefrontal cortex (mPFC) and the basolateral amygdala (BLA), and the expression of fear involves synchronized activity in θ and γ oscillatory activities. In addition, freezing, the most classical measure of fear response in rodents, temporally coincides with the development of sustained 4-Hz oscillations in prefrontal-amygdala circuits. Interestingly, these oscillations were recently shown to depend on the animal’s respiratory rhythm, supporting the growing body of evidence pinpointing the influence of nasal breathing on brain rhythms. During fearful states, rats also emit 22-kHz ultrasonic vocalizations (USVs) which drastically affect respiratory rhythm. However, the relationship between 22-kHz USV, respiration, and brain oscillatory activities is still unknown. Yet such information is crucial for a comprehensive understanding of how the different components of fear response collectively modulate rat’s brain neural dynamics. Here, we trained male rats in an odor fear conditioning task, while recording simultaneously local field potentials (LFPs) in BLA, mPFC, and olfactory piriform cortex (PIR), together with USV calls and respiration. We show that USV calls coincide with an increase in delta and gamma power and a decrease in theta power. In addition, during USV emission in contrast to silent freezing, there is no coupling between respiratory rate and delta frequency, and the modulation of fast oscillations amplitude relative to the phase of respiration is modified. We propose that sequences of USV calls could result in a differential gating of information within the network of structures sustaining fear behavior, thus potentially modulating fear expression/memory.

## Visual Abstract

**Figure:**
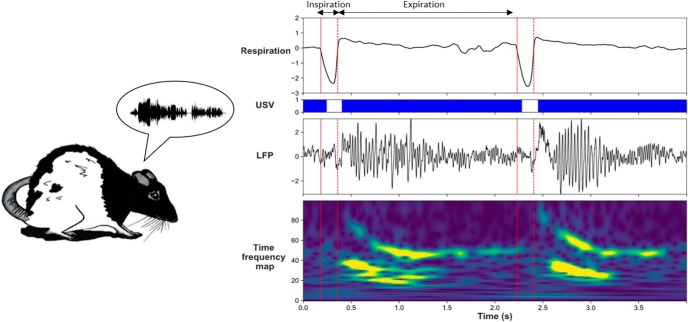

## Significance Statement

While freezing is the most frequently used measure of fear, it is only one among the different components of rodents’ response to threatening events. Ultrasonic vocalization (USV) is another index that gives additional insight into the socioemotional status of an individual. Our study is the first to describe the effects of USV production on rat’s brain oscillatory activities in the fear neural network, and to relate some of them to changes in nasal breathing. A better knowledge of the impact of social vocalizations on brain neural dynamics is not only important for understanding the respective weight of the different components of fear response, but is also particularly relevant for rodent models of human neuropsychiatric disorders, for which socio-affective communication is severely impaired.

## Introduction

Fear behavior has been shown to depend on the interaction between the median prefrontal cortex (mPFC) and the basolateral amygdala (BLA), and to involve synchronized activity in theta (4–12 Hz) and gamma (30–120 Hz) frequency oscillations (Seidenbecher et al., 2003; Popa et al., 2010; Headley and Paré, 2013; Herry and Johansen, 2014; Likhtik et al., 2014; Stujenske et al., 2014; Bocchio et al., 2017). In addition, recent studies have shown that freezing, the most used index of fear response in rodents, temporally coincides with the development of sustained 4-Hz oscillations causally involved in the synchronization of spiking activity between prelimbic mPFC and amygdala (Dejean et al., 2016; Karalis et al., 2016). Importantly, this slow oscillation is distinct from the theta rhythm and predicts the onset and offset of freezing. Interestingly, recent work has shown that freezing-related 4-Hz oscillation in the prelimbic mPFC was correlated with the animal’s respiratory rate, and that disruption of olfactory inputs to the mPFC significantly reduces the 4-Hz oscillation in this structure (Moberly et al., 2018). These data bring further support to the growing body of evidence showing that in addition to its impact on olfactory regions (for review, see Buonviso et al., 2006), nasal respiration also entrains oscillations in widespread brain regions including those involved in the fear network like the mPFC and amygdala (for review, see Tort et al., 2018a). This suggests that the breathing rhythm, akin to slow oscillatory rhythms, could help coordinate neural activity across distant brain regions (Jensen and Colgin, 2007; Heck et al., 2017), and potentially modulate emotional/cognitive processes.

Freezing is only one among the different components of rodents’ response to a threatening event. In aversive situations, such as exposure to predator or foot-shock, rats also emit 22-kHz ultrasonic vocalizations (USVs; Schwarting and Wöhr, 2012). They indicate a negative emotional state and are associated with the termination of social behavior and the avoidance of social contacts. Their study provides a powerful tool to assess emotionality and social behavior in animal models of pathologies like autism (Wöhr and Scattoni, 2013). Surprisingly, to our knowledge no study has assessed the impact of USV production on the animal’s brain neural dynamics. Yet such information is crucial to understand how the different components of fear response collectively modulate rat’s brain neural dynamics. Indeed the emission of USV is considered as reflecting a change in emotional level and 22-kHz USV rates increase with the aversiveness of the situation, as evidenced when foot-shock intensity is increased (Wöhr et al., 2005; Hegoburu et al., 2011). Importantly, 22-kHz USV emission drastically slows down the animal’s respiratory rate (Frysztak and Neafsey, 1991; Hegoburu et al., 2011; Sirotin et al., 2014; Boulanger-Bertolus et al., 2017), potentially disrupting the respiratory-related brain rhythm described above. The present study thus aimed (1) to investigate whether USV emission coincides with specific changes in oscillatory activities in the fear neural network, and (2) to assess to what extent these changes are related to changes in respiratory rate.

To do so, rats were trained in an odor fear conditioning paradigm while local field potentials (LFPs) in BLA, mPFC, and olfactory piriform cortex (PIR) were monitored simultaneously with USV calls, behavior and respiration, during the post-shock period. BLA and mPFC were chosen for their well-known role in learned fear acquisition and expression (LeDoux, 2000; Corcoran and Quirk, 2007). The PIR was included as a recording site because it is involved in odor fear conditioning (Sevelinges et al., 2004; Hegoburu et al., 2009, 2014; Sacco and Sacchetti, 2010) and establishes direct connections with both the PFC (Clugnet and Price, 1987) and the amygdala (McDonald, 1998). We report that USV emission temporally coincides with a significant increase in delta, beta, and gamma activities while a decrease in theta activity is observed. In addition, we show that some of these changes co-occur with USV-induced changes in respiration. The present data suggest that USV calls could result in a differential gating of information within the fear neural network, thus potentially modulating fear memory/expression.

## Materials and Methods

### Animals

Data were obtained from twenty-two male Long Evans rats (250–270 g at their arrival, Janvier Labs). They were housed individually at 23°C and maintained under a 12/12 h light/dark cycle (lights on from 8 A.M. to 8 P.M.). Food and water were available ad libitum. All experiments and surgical procedures were conducted in strict accordance with the European Community Council Directive of September 22, 2010 (2010/63/UE) and the national ethics committee (APAFIS#10606). Care was taken at all stages to minimize stress and discomfort to the animals.

### Surgery

Animals were anesthetized with Equithesin, a mixture of chloral hydrate (127 mg/kg, i.p.) and sodium pentobarbital (30 mg/kg, i.p.), and placed in a stereotaxic frame (Narishige) in a flat skull position. The level of anesthesia was held constant with regular injections of Equithesin throughout the experiment. Monopolar stainless steel recording electrodes (100 µm in diameter) were then stereotaxically implanted in the left hemisphere in the three brain areas: PIR (AP: –1.8 mm, L: +5.5 mm, DV: –8 mm), mPFC (AP: +3.0 mm; L: +0.8 mm; DV: –3.5 mm), and BLA (AP: –2.8 mm, L: +4.9 mm, DV: –7.5 mm). Accurate positioning in the PIR was achieved using the characteristic profiles of evoked field potential induced in the PIR in response to electrical stimulation of the olfactory bulb (Haberly, 1973). For this, a bipolar stimulation electrode (made of two 100-μm stainless-steel wires with a tip separation of 500 μm) was lowered transiently in the olfactory bulb to facilitate positioning in the PIR and withdrawn thereafter. A reference electrode was screwed in the skull above the right parietal lobe. The three recording electrodes were connected to a telemetry transmitter (rodentPACK system, EMKA Technologies) fixed to the rat’s skull surface by dental acrylic cement and anchored with a surgical screw placed in the frontal bone. The animals were allowed to recover for two weeks following surgery.

### Experimental apparatus

The apparatus has been described in detail in a previous study (Hegoburu et al., 2011). It consisted of a whole-body customized plethysmograph (diameter 20 cm, height 30 cm, EMKA Technologies) placed in a sound-attenuating cage (length, 60 cm; width, 60 cm; height, 70 cm, 56-dB background noise). The plethysmograph was used to measure respiratory parameters in behaving animals. The ceiling of the plethysmograph was equipped with a tower allowing the introduction of a condenser ultrasound microphone (Avisoft-Bioacoustics CM16/CMPA) to monitor USVs emitted by the rats. The bottom of the animal chamber was equipped with a shock floor connected to a programmable Coulbourn shocker (Bilaney Consultants GmbH). Three Tygon tubing connected to a programmable custom olfactometer were inserted in the tower on the top of the plethysmograph to deliver air and odorants. Deodorized air flowed constantly through the cage (2 l/min). When programmed, an odor (McCormick Pure Peppermint; 2 l/min; 1:10 peppermint vapor to air) was introduced smoothly in the air stream through the switching of a solenoid valve (fluid automation systems, CH-1290 Versoix), thus minimizing its effect on change in pressure. The bottom of the animal chamber had a port connected to a ventilation pump which could draw air out of the plethysmograph (at a rate of up to 2 l/min) thus maintaining a constant airflow that did not interact with the animal’s breathing pattern. Animal’s behavior was monitored with two video cameras on the walls of the sound-attenuating cage.

### Fear conditioning paradigm and data acquisition

After the recovery period, the animals were handled individually and placed in the experimental apparatus for 30 min each day during 3–4 d before the beginning of the experiments to familiarize them with being manipulated and connected to the telemetry transmitter.

For the conditioning session, the telemetry transmitter was plugged on the animal’s head and the rat was allowed free exploration during the first 4 min, then an odor was introduced into the cage for 20 or 30 s, the last second of which overlapped with the delivery of a 0.4-mA foot-shock. The animal received 10 odor-shock trials, with an intertrial interval of 4 min. After the last pairing, the transmitter was unplugged and the animal returned to its home cage.

### Retention test

The conditioned fear response was assessed during a retention test conducted 48 h after conditioning. For the retention test, the rat was placed in the experimental cage (equipped with new visual cues and with a plastic floor to avoid contextual fear expression) and allowed a 4-min odor-free period. The CS odor was then presented five times for 20 s with a 4-min intertrial interval. The animal’s freezing response was quantified during each 20-s odor presentation and averaged across the five trials.

### Data acquisition and preprocessing

For USV recording, the ultrasound microphone was connected to a recording interface (UltraSoundGate 116 Hb, Avisoft-Bioacoustics) with the following settings: sampling rate = 214,285 Hz; format = 16 bit (Wöhr et al., 2005). Recordings were transferred to Avisoft SASLab Pro (version 4.2, Avisoft-Bioacoustics) and a fast Fourier transform (FFT) was conducted. Spectrograms were generated with an FFT length of 512 points and a time window overlap of 87.5% (100% Frame, FlatTop window). These parameters produced a spectrogram at a frequency resolution of 419 Hz and a time resolution of 0.29 ms. The acoustic signal detection was provided by an automatic whistle tracking algorithm with a threshold of −20 dB, a minimum duration of 0.01 s and a hold time of 0.02 s. However, the accuracy of detection was verified trial by trial by an experienced user. The main parameters used in the present study were extracted using Avisoft SASLab Pro and concerned the duration as well as the peak amplitude and peak frequency of USV calls. No band pass filter has been applied during USV recording. Although a few 50-kHz USVs were observed following shock delivery, in the present study, we focused on 22-kHz USV.

The respiratory signal collected from the plethysmograph was amplified and sent to an acquisition card (MC-1608FS, Measurement Computing; sampling rate = 1000 Hz) for storage and offline analysis. The detection of the respiratory cycles was achieved using an algorithm described in a previous study (Roux et al., 2007). This algorithm performs two main operations: signal smoothing for noise reduction, and detection of zero-crossing points to define accurately the inspiration and expiration phase starting points. Momentary respiratory frequency was determined as the inverse of the respiratory cycle (inspiration plus expiration) duration.

The video signals collected through the two cameras were transmitted to a video acquisition card and a homemade acquisition software. Offline, freezing behavior defined as the absence of any visible movement except that due to breathing (Blanchard and Blanchard, 1969), was automatically detected using a Labview homemade software and further verified by an experimenter. For this, on each video recording, two successive images were subtracted and the resulting image was binarized using a gray level threshold. The pixels below this threshold were encoded in black and those above the threshold in white. The absence of white pixels on the image resulted in the scoring of freezing state. Then the same analysis was conducted for the next images of the video recording. The animal’s freezing behavior was thus analyzed with a 40-ms (two consecutive images) time bin. As a final step, in accordance with classical encoding of freezing behavior in the literature, only freezing episodes lasting longer than 1 s were considered as stable freezing behavior. Behaviors other than freezing were manually checked and classified. Escape attempt was scored when the animal exhibited wall climbing, running, or saccadic head movements. Although the latter do not involve directed locomotion due to the small size of the plethysmograph, they are clearly different from active exploratory behavior. They might have been induced by the small size of our cage and the absence of any escape route.

LFPs were collected by telemetry via a three-channel wireless miniature transmitter (<5.2 g, RodentPack EMKA Technology). LFP signals were amplified (1000×), filtered (between 0.1 and 100 Hz), digitized (sampling frequency = 1000 Hz), and stored on a computer for offline analysis.

### Data analysis

#### Data selection and experimental categories

**Figure 1.**
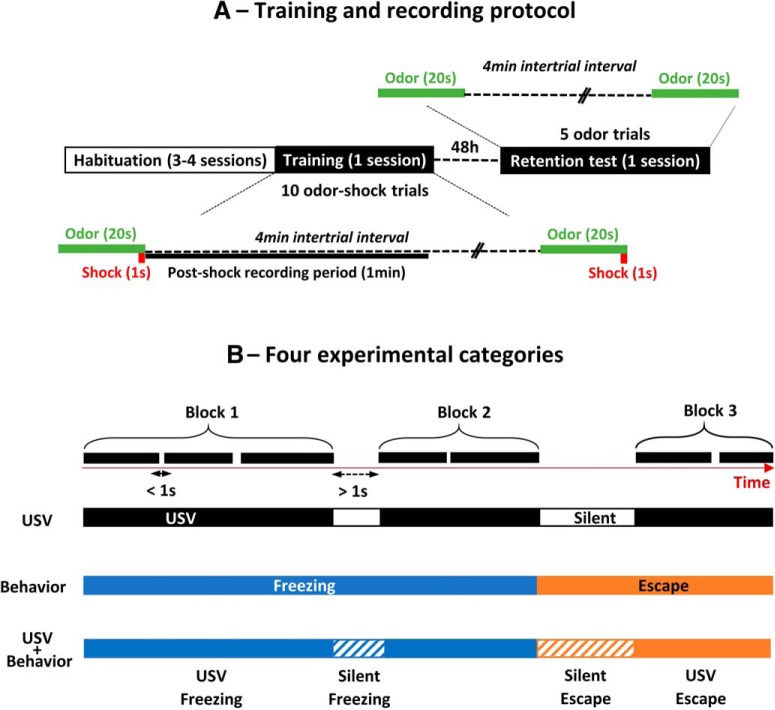
***A***, Training and recording protocol. The animals were trained with 10 odor (20 s)-shock (1 s) pairings. During the 1-min post-shock period, LFPs were recorded together with USVs and behavior; 48 h later, a retention test was conducted using five odor (20 s) presentations during which the animal’s freezing response was assessed. ***B***, Definition of four experimental categories for data analysis. During the 1-min post-shock period, we defined blocks of USV corresponding to successive USV with less than 1 s between each other. When the interval between two USV exceeded 1 s, then a new block was defined (first row). The periods between USV blocks are considered as silent periods. In parallel, the synchronized animal’s behavior (freezing or escape) was detected, and four different combinations were obtained: USV freezing, USV escape, silent freezing, and silent escape. For each combination, only segments longer than 1 s were considered.

Since the aim of the study was to assess the relationship between USV emission, respiration, and brain oscillatory activity, we focused our analysis on the 1-min period following shock delivery during which USVs were numerous and loud. During this period, the animal’s behavior was of two types: freezing or escape attempt. No other type of behavior (like grooming, exploration, quiet immobility…) was observed. We first noticed that while the majority of USVs were emitted during freezing, a substantial amount of USV also occurred during escape behavior. This led us to distinguish four types of experimental categories: silent freezing, USV freezing, silent escape, and USV escape (Fig. 1). For each category, only segments longer than 1 s were considered for further analysis.

#### LFP signals spectral analysis

The different data (respiration, USV, behavior, LFP signals) were synchronized offline via a TTL synchronization signal generated at the beginning of each experimental session. Once synchronized, the data were analyzed using custom-written scripts under Python.

The LFP signals were first individually inspected to eliminate artifacts due to signal saturation or transient signal loss. The selection was made for each recording site separately and proceeded as follows: when the duration of an artifact exceeded 5 s over the 60-s post-shock recording, the trial was excluded. When the number of excluded trials exceeded five (out of the 10 trials), then the recording site was excluded for this animal. This procedure led to the following number of animals per recording site for all the electrophysiological data: BLA, *n* = 14; CPF, *n* = 21; PIR, *n* = 20. Because the average duration of individual USV calls was too short to allow proper oscillatory activity analysis (notably for the slow oscillations), we conducted the analysis on blocks of USV corresponding to successive USV with less than 1 s between each other (Fig. 1). As soon as the interval between two USV exceeded 1 s, then a new block was defined. The periods between USV blocks are considered as silent periods.

The power spectral density (PSD) of the LFP signals was calculated using the continuous Morlet wavelet transform (Kronland-Martinet et al., 1987) instead of the classical windowed Fourier transform. Indeed, the continuous wavelet transform is less susceptible to non-stationary events and offers a better time–frequency resolution. The Morlet wavelet estimated the amplitude of the signal at each time and frequency bin. The obtained time frequency map was then segmented in periods of interest with variable durations (corresponding to the four above defined experimental categories) and averaged per category. Four frequency bands were identified for the subsequent analyses: delta (0–5 Hz), theta (5–15 Hz), beta (15–40 Hz), and gamma (40–80 Hz) and the mean power in the different frequency bands was calculated. The values obtained for each recording site were averaged across animals.

#### Covariation of LFP slow (delta and theta) oscillatory frequency and respiratory frequency

To study frequency-frequency coupling between LFP signals and respiration, we did not use classical coherence analysis because the respiratory signal is not always sinusoidal (especially during USV calls, see Fig. 5*A*). We therefore designed a homemade method allowing to track instantaneous frequency synchrony. To do so, for each detected respiratory cycle, the frequency was estimated as 1/cycle duration and the time course of the instantaneous respiratory frequency was extracted. In parallel, the continuous Morlet scalogram for the LFP signal was computed in our frequency band of interest (0–15 Hz). At each time bin (4 ms), the local maximum in the instantaneous power spectrum was extracted together with the corresponding instantaneous frequency of the LFP signal. The time course of the predominant instantaneous frequency curve of the LFP was then extracted. From the two times series obtained (instantaneous respiration frequency and predominant instantaneous LFP frequency), a 2D matrix histogram was built, with the respiratory frequency represented on the *x*-axis and the LFP frequency on *y*-axis. This 2D histogram was normalized so that the total sum is 1, and point density was represented on a color scale ranging from blue to yellow as the point density increases. The existence of a coupling between respiration frequency and LFP frequency can be assumed when a high point density (i.e., yellow color) is observed along the diagonal of the 2D histogram (see Fig. 6 for an illustration). Conversely, in the absence of coupling a non-correlated Gaussian shape is observed. The two possibilities can co-occur on the same 2D histogram.

#### Modulation of LFP beta and gamma power by respiratory cycle phase

To investigate whether LFP beta and gamma amplitudes were modulated by respiration phase, we computed a so called “cycle-frequency scalogram” of the LFP signal (adapted from Roux et al., 2007). In analogy to a classical time-frequency map that computes the energy of LFP signal over time and frequency, we computed the energy of LFP signal over respiratory cycle duration and frequency. Briefly this method consists in three steps: (1) compute the continuous Morlet scalogram (time-frequency map), (2) use detected respiratory cycle to segment this scalogram in two phases (inhalation, exhalation), and (3) stretch by linear interpolation each segment so that all the segments fit the same normalized template (range from 0 to 1, with a 0.025 bin). The result of this analysis is very similar to the classical time frequency scalogram except that it presents small time distortions locally that do not affect the instantaneous power. Since all the cycles were normalized to the same size, they were averaged together. The typical template of an individual respiratory cycle is defined as follows: inhalation from 0 to 0.4 and exhalation from 0.4 to 1. This ratio corresponds to the average value calculated over all cycles and animals. On the scalogram, LFP signal power for the different frequency bands is represented using a color scale ranging from blue to yellow as the power increases (see Fig. 7*B* for an illustration). The maximum power in the beta and gamma bands was also extracted and represented on a curve throughout the respiratory cycle.

### Statistical analysis

All analyses were performed with Systat 13.0 software. For each test, the significance level was set at *p* < 0.05.

**Figure 2.**
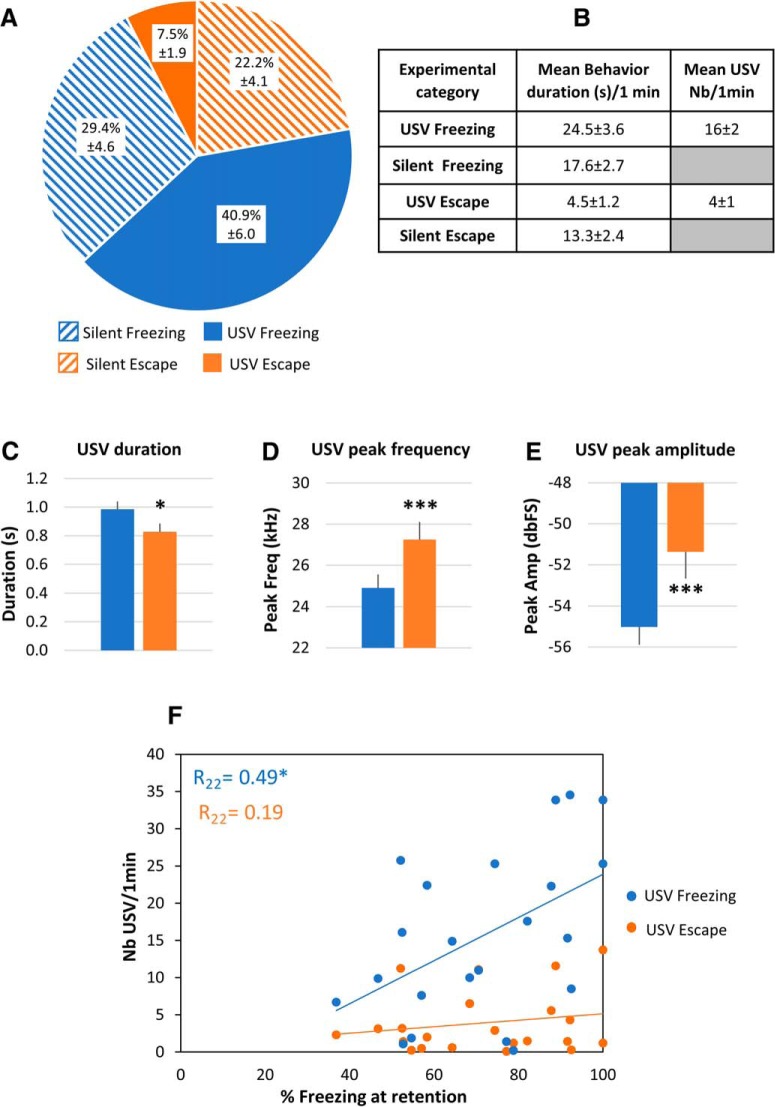
Repartition of the four defined categories and characterization of two 22-kHz USV types (*n* = 22 rats). ***A***, Mean (±SEM) proportion of each category per animal over the 1-min post-shock analysis period. ***B***, Mean (±SEM) duration of the different categories and mean (±SEM) number of USV freezing and USV escape emitted during the 1-min post-shock period. ***C***, Mean duration (±SEM) of the two USV subtypes. ***D***, Mean frequency (±SEM) of the two USV subtypes. ***E***, Mean intensity (±SEM) of the two USV subtypes; *n* = 22 rats, *p < 5 × 10^−2^, ***p < 5 × 10^−3^. ***F***, Correlation between the mean number of USV calls recorded during the 1-min post-shock period at training and the freezing score obtained during the retention test in response to the learned odor; *p < 5 × 10^−2^.

USV parameters (Fig. 2; duration, peak amplitude, peak frequency) were compared between USV freezing and USV escape using paired *t* tests.

**Figure 3.**
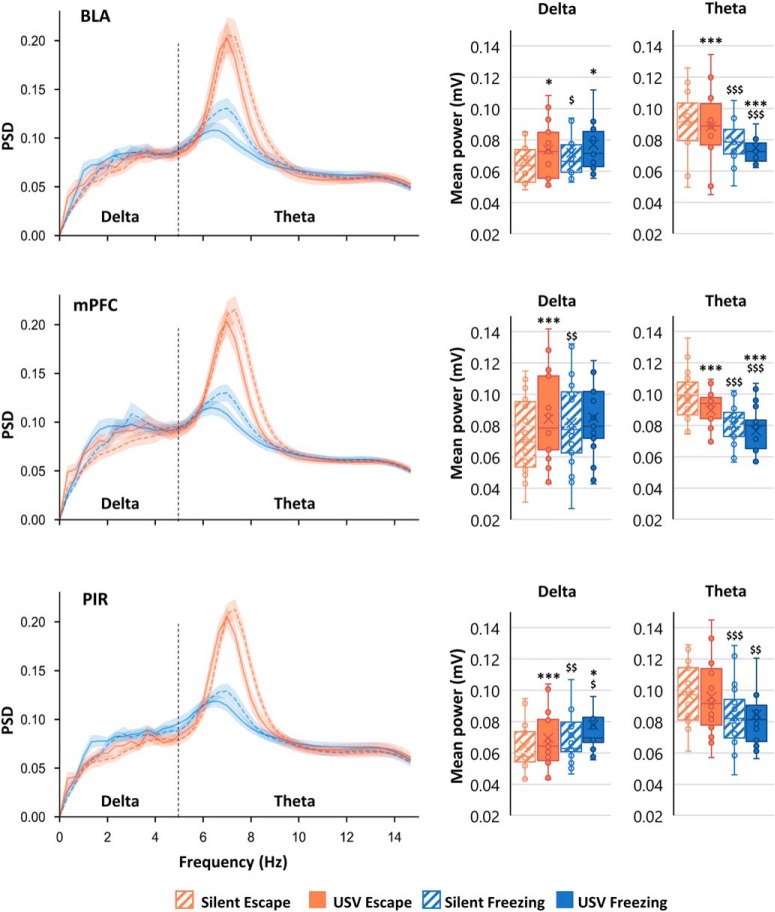
PSD of LFP signals and mean power in delta (0–5 Hz) and theta (5–15 Hz) bands. The average PSD (±SEM) is represented on the left part of the figure, and the delta and theta average power (±SEM) is represented on the right part. BLA: *n* = 14; mPFC: *n* = 21; and PIR: *n* = 20; *p < 5 × 10^−2^, **p < 5 × 10^−3^, ***p < 5 × 10^−4^: significant difference between same color-different pattern bars; $*p* < 5 × 10^−2^, $$*p* < 5 × 10^−3^, $$$*p* < 5 × 10^−4^: significant difference between same pattern-different color bars.

**Figure 4.**
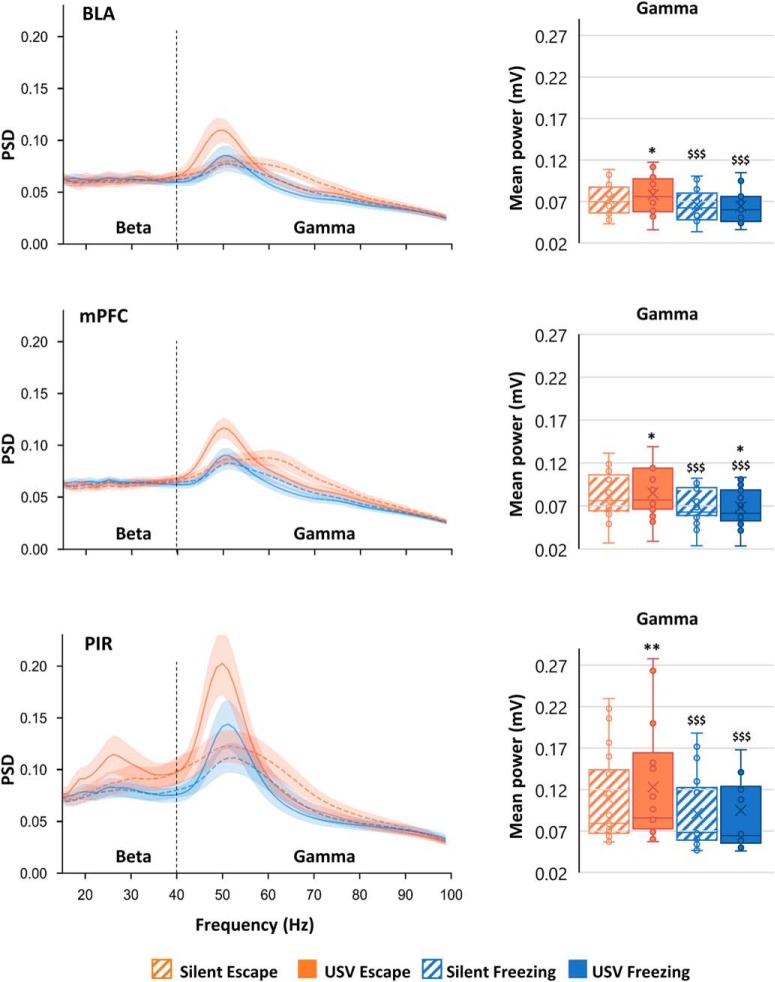
PSD of LFP signals and mean power in gamma (40–80 Hz) band. The average PSD (±SEM) is represented on the left part of the figure, and the gamma average power (±SEM) is represented on the right part. BLA: *n* = 14; mPFC: *n* = 21; and PIR: *n* = 20; *p < 5 × 10^−2^, **p < 5 × 10^−3^, ***p < 5 × 10^−4^: significant difference between same color-different pattern bars; $*p* < 5 × 10^−2^, $$*p* < 5 × 10^−3^, $$$*p* < 5 × 10^−4^: significant difference between same pattern-different color bars.

Average LFP oscillatory activity parameters (Figs. 3, 4; power, peak amplitude, frequency) are calculated in the different frequency bands: delta (0–5 Hz), theta (5–15 Hz), beta (15–40 Hz), and gamma (40–80 Hz), and expressed as mean ± SEM across animals. A three-way (structure, USV, and behavior) ANOVA for repeated measures was first applied to assess between structures differences in the four experimental categories defined above. Then, for each structure, a two-way (USV and behavior) ANOVA for repeated measures was applied followed by *post hoc* multiple comparisons.

The time course of beta and gamma activity power throughout the respiratory cycle (Figs. 8, 9) was first compared using a three-way ANOVA (behavior, USV, and respiratory cycle time), followed by a two-way ANOVA for repeated measures (USV and respiratory cycle time) conducted separately for freezing and escape condition.

### Histology

At the end of the experiment, the animals were killed with a lethal dose of pentobarbital, their brains were removed, postfixed, and cryoprotected in sucrose (20%). The brains were then sectioned (40-µm coronal slices) for verification of electrodes tips by light microscopy. Areas targeted by the electrodes in the three implanted brain regions have been reported on brain atlas coronal sections (Extended Data Fig. 1-1).

10.1523/ENEURO.0065-19.2019.f1-1 Extended Data Figure 1-1 Areas targeted by the electrodes (light orange areas) in the three recording sites. Numbers at the bottom indicate the relative position of coronal slices from bregma (adapted from Paxinos and Watson, 2007). mPFC: *n* = 21; PIR: *n* = 20; and BLA: *n* = 14. Download Figure 1-1, TIF file.

## Results

### 22-kHz USVs are observed during both passive and active defense responses

The 1-min period following shock delivery was analyzed for behavior, USV emission and brain oscillatory activity. During this period, the animal’s behavior was of two types: freezing or escape attempts. While the majority of USVs were emitted during freezing, a non-negligible amount of USV also occurred during escape. Figure 2*A* illustrates the repartition of the four categories throughout the 1-min post-shock period. The animals spent 70.3% of the time in freezing versus 29.7% in escape. While the animals spent similar amounts of time in silent freezing compared to USV freezing, they spent more time in silent escape than in USV escape. Figure 2*B* reports the mean behavior duration in the four conditions and the mean USV rate during the 1-min post-shock period.

We then compared the characteristics of the USV emitted during freezing versus escape. Paired *t* test comparisons revealed that call duration (Fig. 2*C*) was significantly lower for USV emitted during escape than during freezing (*t*_(21)_ = 2.198, *p* = 0.039), while call peak amplitude (Fig. 2*D*) and peak frequency (Fig. 2*E*) was significantly higher during escape than during freezing (peak amplitude: *t*_(21)_ = –3.957, *p* = 0.001; peak frequency: *t*_(21)_ = –3.928, *p* = 0.001).

Finally, we assessed whether the amount of USV (USV freezing or USV escape) emitted during conditioning could predict the animal’s performance during the retention test conducted 48 h later (Fig. 2*F*). We showed that the number of USV freezing was positively correlated with the amount of freezing at retention (Pearson correlation coefficient: R_22_ = 0.49, *p* < 0.02) while the number of USV escape was not (R_22_ = 0.19).

In summary, 22-kHz USVs are emitted during both passive (freezing) and active (escape) defense responses. USV emitted during escape are shorter and louder than those emitted during freezing, and exhibit a higher peak frequency. In addition, the amount of USV freezing during training was a good predictor of the animal’s learned fear response at retention.

### USV emission is associated with changes in oscillatory activity power

The main objective of this experiment was to assess whether USV emission is associated with changes in oscillatory activity power compared to the silent behavioral state, and if these changes are similar across the three recording sites. Because USVs are emitted during two different defense responses, we also compared oscillatory activity power between these two behavioral states (freezing vs escape).

### Delta (0–5 Hz) and theta (5–15 Hz) bands mean PSD

For each recording site, LFP mean PSD was calculated per animal and averaged across animals (Fig. 3, left part). It can be observed that in each recording site, the signal power in the delta and theta bands depends both on the animal’s behavioral state (freezing vs escape) and for a given behavioral state, on the emission of USV (USV vs silent). Averaged mean power was calculated for each frequency band, in the four categories.

#### Delta Band mean power (Fig. 3, middle column)

A three-way ANOVA revealed no main effect of structure (*F*_(2,52)_ = 1.38, *p* = 0.3), but a significant USV × behavior × structure interaction (*F*_(2,52)_ = 3.23, *p* = 0.05). In each recording sites, a two-way ANOVA revealed a significant main effect of factors USV and behavior, and no significant USV × behavior interaction except for the mPFC (see Extended Data Fig. 3-1 for all the statistical results). *Post hoc* comparisons first showed that in the three structures, δ mean power was higher during silent freezing than during silent escape. In addition, in both behavioral states (except for mPFC for which the effect of USV was only significant during escape), the emission of USV was associated with a significant enhancement in delta mean power.

10.1523/ENEURO.0065-19.2019.f3-1 Extended Data Figure 3-1 Delta and Theta activity mean power statistical data: ANOVA analysis (upper table) and p values for post-hoc comparisons (lower table). * : p≤5x10^-2^, ** : p≤5x10^-3^, *** : p≤5x10^-4^. Download Figure 3-1, DOCX file.

#### Theta Band mean power (Fig. 3, right column)

The three-way ANOVA revealed no main effect of structure (*F*_(2,52)_ = 0.38, *p* = 0.7), but a significant USV × structure interaction (*F*_(2,52)_ = 4.72, *p* = 0.01). In BLA and mPFC, the two-way ANOVA revealed a significant main effect of factors USV and behavior, and no significant USV × behavior interaction (Extended Data Fig. 3-1, upper part). In PIR, no significant effect of factor USV was observed. *Post hoc* comparisons (Extended Data Fig. 3-1, lower part) showed that in the three structures, theta mean power was lower during silent freezing than during silent escape. Moreover, in BLA and mPFC, USV emission was associated with a decrease in theta mean power.

In summary, when compared to silent escape, silent freezing is characterized by a higher power of oscillatory activity in the delta band while a lower power was observed in the theta band. Importantly, in both freezing and escape states, the emission of USV coincides globally with an increase in power in the delta band in all three regions and a decrease in the theta band in BLA and mPFC.

### Beta (15–40 Hz) and gamma (40–80 Hz) bands mean PSD

Mean PSD of LFPs is represented for each recording site in Figure 4, left part. It can be observed that in each recording site, the signal power in the gamma band depends on both the animal’s behavioral state (freezing vs escape) and, for a given behavioral state, the emission of USV (USV vs silent). Furthermore, in the PIR, the signal power in the beta band also seems to be affected during the emission of USV during escape. Averaged mean power was calculated for each frequency band, in the four categories.

#### Beta Band mean power (Extended Data Fig. 4-1)

Beta Band mean power values in the three recording sites are reported on Extended Data Fig. 4-1, upper part. The three-way ANOVA revealed a significant main effect of Structure (*F*_(2,52)_ = 4.24, *p* = 0.02), and a significant interaction for USV × structure (*F*_(2,52)_ = 4.10, *p* = 0.02), behavior × structure (*F*_(2,52)_ = 5.15, *p* = 0.009) and USV × behavior × structure (*F*_(2,52)_ = 5.32, *p* = 0.008). The two-way ANOVA revealed no significant changes in beta activity in the BLA (Extended Data Fig. 4-1, middle part). In the mPFC, a significant main effect of USV was observed, and *post hoc* comparisons showed that USV emission during both Escape and Freezing induced an increase in β mean power (Extended Data Fig. 4-1, lower part). In the PIR, a significant main effect of USV and behavior, and a significant USV × behavior interaction were observed. *Post hoc* comparisons showed that USV emission during escape was associated with an increase in beta mean power.

10.1523/ENEURO.0065-19.2019.f4-1 Extended Data Figure 4-1 Beta band mean power values (+/- sem) in the three recording sites (upper table), ANOVA analysis (middle table), and p values for post-hoc comparisons (lower table). * : p≤5x10^-2^, ** : p≤5x10^-3^, *** : p≤5x10^-4^. Download Figure 4-1, DOCX file.

#### Gamma Band mean power (Fig. 4, right column)

The three-way ANOVA revealed a significant main effect of structure (*F*_(2,52)_ = 3.69, *p* = 0.02), and a significant interaction for USV × structure (*F*_(2,52)_ = 4.20, *p* = 0.02) and behavior × structure (*F*_(2,52)_ = 5.29, *p* = 0.008). The two-way ANOVA revealed a significant main effect of behavior, and a significant USV × behavior interaction in the three recording sites (Extended Data Fig. 4-2). *Post hoc* comparisons showed that gamma mean power was lower during silent freezing than during silent escape. In addition, USV emission during escape temporally coincided with an increase in gamma mean power. Noteworthy, USV emission during both freezing and escape was associated with a narrowing of the activity toward the lower range of the band and an increase in gamma peak power (as can be seen on the power spectra of Fig. 4, left column).

10.1523/ENEURO.0065-19.2019.f4-2 Extended Data Figure 4-2 Gamma band mean power statistical data in the three recording sites: ANOVA analysis (upper table) and p values for post-hoc comparisons (lower table). * : p≤5x10^-2^, ** : p≤5x10^-3^, *** : p≤5x10^-4^. Download Figure 4-2, DOCX file.

In summary, during silent freezing, gamma band mean power is lower than during silent escape. In addition, the emission of USV coincides with an increase in gamma band activity (mainly during escape) added to a narrowing of the activity toward the lower range of the band where the peak power is increased. Finally, in the PIR and mPFC (but not in BLA), the emission of USV during escape is associated with an increase in beta activity power.

### USV emission strongly affects instantaneous respiratory rate

**Figure 5.**
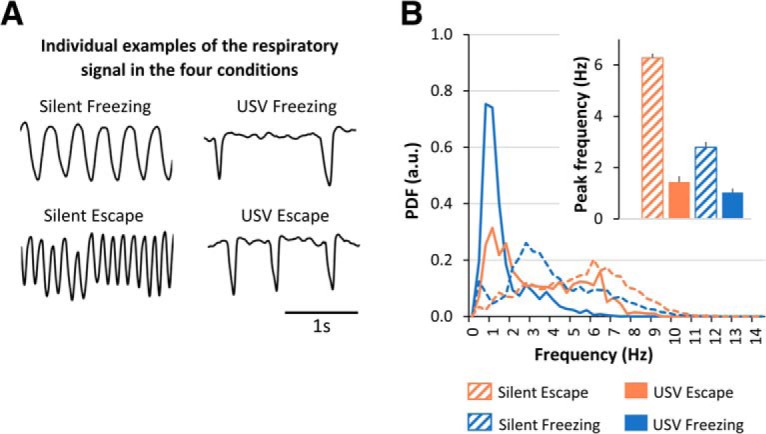
Characterization of respiratory frequency in the four experimental categories (*n* = 22 rats). ***A***, Individual examples of the respiratory signal. ***B***, PDF of respiratory frequency. The distributions were obtained using a 0.33-Hz bin. Inset, Average peak frequency (±SEM) in each category.

As previously reported in the literature, we found that the emission of USV drastically changes the shape and frequency of the respiratory signal (see individual examples in Fig. 5*A*). Figure 5*B* illustrates the probability distribution function (PDF) of respiration in our four experimental categories. A three-way ANOVA revealed a highly significant behavior × USV × respiratory frequency interaction (*F*_(32,1344)_ = 5.44, *p* < 0.000001) and further two-way ANOVAs showed a significant main effect of USV for both freezing (*F*_(1,21)_ = 6.29, *p* = 0.02) and escape (*F*_(1,21)_ = 18.05, *p* = 0.0004) states. The emission of USV shifts the dominant respiratory frequency toward lower values, going from 6.3 to 1.4 Hz for escape and from 2.8 to 1 Hz for freezing (Fig. 5*B*, inset). The next step of our study was then to assess to what extent the frequency of oscillatory activity in the delta and theta range followed respiratory frequency, and consequently whether USV emission has an impact on this relationship.

### Covariation between delta and theta oscillatory frequencies and respiratory frequency

**Figure 6.**
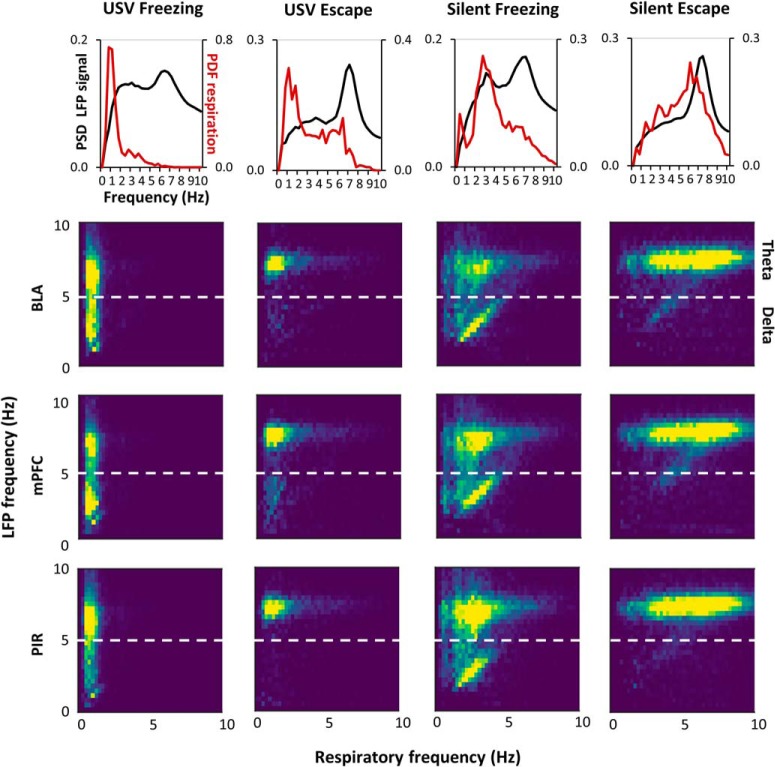
Covariation between delta and theta oscillatory frequencies and respiratory frequency. ***A***, Each graph represents the PSD of LFP signals (left *y*-axis, black curve) and the PDF of respiration (right *y*-axis, red curve). The graphs were obtained from LFP signals recorded in the mPFC in the four experimental categories (silent escape, USV escape, silent freezing, and USV freezing). ***B***, 2D matrix histograms obtained from LFP signals recorded in mPFC (*n* = 21), BLA (*n* = 14), and PIR (*n* = 20), *y*-axis represents LFP frequency and *x*-axis respiratory frequency. The 2D histogram is normalized so that the total sum is 1, and point density is represented on a color scale ranging from blue to yellow as the point density increases.

Figure 6, upper part, highlights the fact that the respiratory frequency range delimits the range of LFP oscillatory frequency for which co-variation between the two signals frequency could be assessed.

In each experimental category, we conducted covariation matrices (Fig. 6, lower part) depicting pairwise similarities between respiratory frequency and oscillatory frequency in the delta and theta bands. During silent escape and USV escape, theta activity is preferentially expressed and shows no obvious coupling with respiration: whatever the respiratory frequency, theta activity is mostly observed with a fixed frequency around 6–7 Hz. During silent freezing, both theta and delta activities are expressed. While a clear-cut covariation is observed between delta frequency and respiratory frequency, no covariation is seen for theta frequency. During USV freezing, both theta and delta activities are expressed with no coupling with respiratory frequency. Raw signal traces recorded in the same animal in the four experimental categories are reported on Extended Data Figure 6-1.

10.1523/ENEURO.0065-19.2019.f6-1 Extended Data Figure 6-1 Examples of raw traces obtained in the same animal in the three recording sites and the four experimental categories. Each panel represents from the top: USVs calls (for the panels on the right), raw respiratory signal, and LFP signals recorded in the BLA, mPFC, and PIR. Download Figure 6-1, TIF file.

In summary, the only experimental category leading to a clear-cut frequency-frequency coupling between respiration and LFP signal is silent freezing and concerns the δ band. During USV emission, no covariation is observed between respiratory rate and delta or theta oscillatory activities.

### Modulation of beta and gamma power with the phase of the respiratory cycle

**Figure 7.**
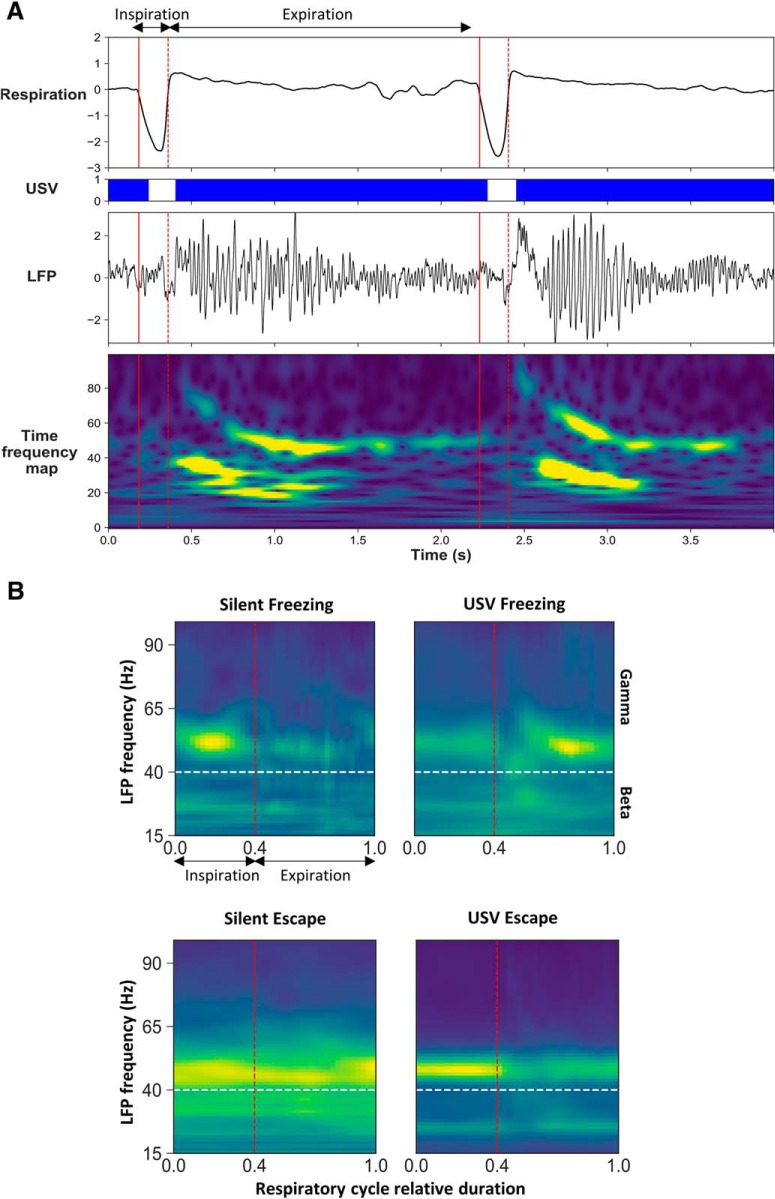
Modulation of beta and gamma power by the phase of the respiratory cycle. ***A***, Individual traces representing from the top, respiratory signal, USV calls, raw LFP signal recorded in the PIR and its time frequency map (*y*-axis: LFP signal frequency in Hz, *x*-axis: time in milliseconds). LFP signal power is represented using a color scale going from blue to red as the power increases. The red vertical plain line represents the transition between expiration and inspiration, while the red vertical dotted line represents the transition between inspiration and expiration. ***B***, Average time frequency map centered on the normalized respiratory cycle, in the four experimental categories. The red vertical dotted line represents the transition between inspiration and expiration phase that was set at 0.4 (this value corresponds to the mean ratio between inspiration and expiration over the four experimental categories). The white horizontal dotted line represents the transition between beta and gamma bands.

**Figure 8.**
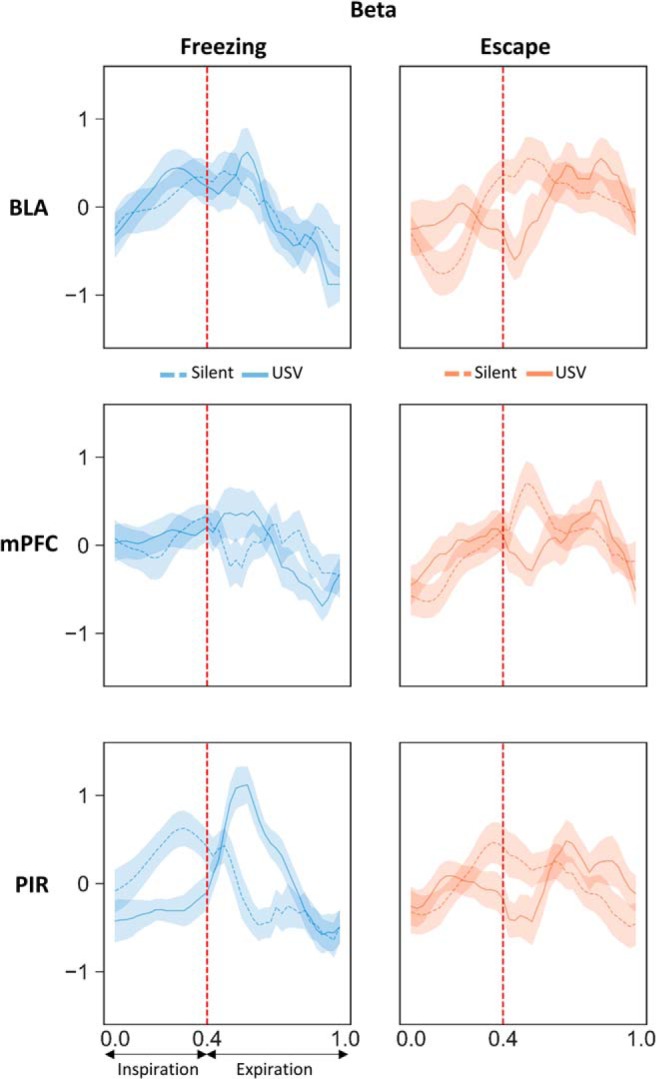
Beta Activity power time course throughout the normalized respiratory cycle in the three recording sites and in the four experimental categories. Left side, Silent freezing versus USV freezing. Right side, Silent escape versus USV escape. The vertical dotted line on each graph represents the transition between inspiration and expiration phase positioned at 0.4 (this value corresponds to the mean ratio between inspiration and expiration over the four experimental categories). BLA: *n* = 14; mPFC: *n* = 21; and PIR: *n* = 20.

**Figure 9.**
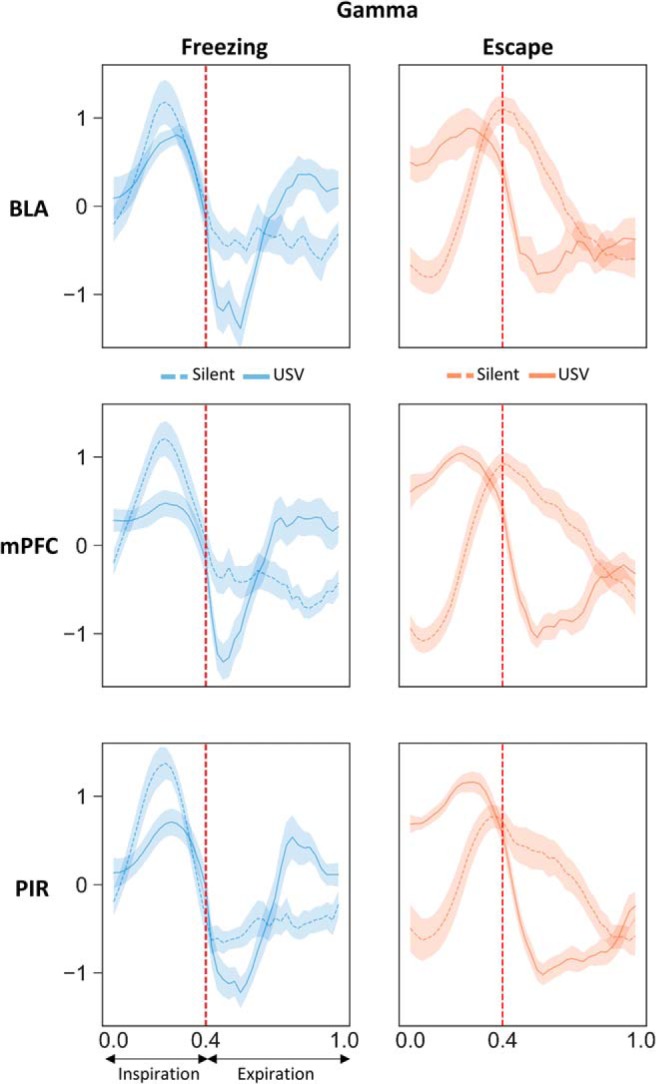
Gamma Activity power time course throughout the normalized respiratory cycle in the three recording sites and in the four experimental categories. Left side: Silent freezing versus USV freezing. Right side, Silent escape versus USV escape. The vertical dotted line on each graph represents the transition between inspiration and expiration phase positioned at 0.4 (this value corresponds to the mean ratio between inspiration and expiration over the four experimental categories). BLA: *n* = 14; mPFC: *n* = 21; and PIR: *n* = 20.

It was shown that respiration can modulate not only slow neuronal oscillations, but also beta and gamma band oscillations, which amplitude is modulated in phase with respiration (Cenier et al., 2009; Ito et al., 2014). We therefore investigated whether activity in the beta and gamma bands was modulated by the phase of the respiratory cycle in our different experimental categories, and whether USV emission has an impact on this modulation. Figure 7*A* illustrates an individual example of LFP signal collected in the PIR, with the corresponding respiratory signal and USV calls. A time frequency analysis conducted on the LFP signal at the level of the respiratory cycle, clearly shows that respiration modulates beta and gamma activity power, with higher beta activity power at the beginning of expiration and higher gamma activity throughout expiration. This modulation is further evidenced by the analysis illustrated in Figure 7*B*, which represents the respiration phase-frequency map of LFP signal in the four experimental categories. We conducted this analysis in the three recording sites (Extended Data Fig. 7-1). The data illustrated in Figure 8 represent the time course of beta activity maximal power throughout the respiratory cycle. A three-way (behavior, respiratory cycle time, USV) ANOVA first revealed a significant main effect of behavior in mPFC (*F*_(1,40)_ = 6.38, *p* = 0.02) and PIR (*F*_(1,38)_ = 7.93, *p* = 0.008), and a significant respiratory cycle time × behavior interaction in BLA (*F*_(39,1014)_ = 2.47, *p* = 2 × 10^−6^). We then analyzed the data separately for freezing and escape to compare the time course of LFP oscillatory activity power throughout the respiratory cycle, either with or without USV. During freezing (Fig. 8, left part), a two-way ANOVA revealed a significant effect of respiratory cycle time in the three recording sites and a significant interaction for USV × respiratory cycle time in the PIR only (Extended Data Fig. 8-1, upper part). In this recording site, during silent freezing, the maximum power is observed during inspiration, while during USV freezing, the maximum is shifted toward the early part of expiration. Concerning escape (Fig. 8, right part), in the three recording sites the ANOVA revealed a significant effect of respiratory cycle time but no effect of USV or USV × respiratory cycle interaction (Extended Data Fig. 8-1, upper part). The time course of gamma activity maximal power throughout the respiratory cycle is reported in Figure 9. The three-way (behavior, respiratory cycle time, USV) ANOVA revealed a significant respiratory cycle time × USV × behavior interaction in BLA (*F*_(39,1014)_ = 0.58, *p* = 1 × 10^−7^), mPFC (*F*_(39,1560)_ = 3.75, *p* = 1 × 10^−7^), and PIR (*F*_(39,1482)_ = 4.18, *p* = 1 × 10^−7^). We then analyzed the data separately for freezing and escape. During freezing (Fig. 9, left part), the two-way ANOVA revealed a significant effect of respiratory cycle time and a significant interaction for USV × respiratory cycle time in the three recording sites (Extended Data Fig. 8-1, lower part). During silent freezing, the maximum power is observed during inspiration, while during USV freezing, two maxima are observed, one during inspiration, and the other during the late part of expiration. Concerning escape (Fig. 9, right part), the ANOVA revealed a significant effect of respiratory cycle time and USV (except for BLA), and a significant interaction for USV × respiratory cycle (Extended Data Fig. 8-1, lower part). During silent escape, the maximum power is observed at the transition between inspiration and expiration, while during USV escape, the maximum is shifted toward inspiration.

10.1523/ENEURO.0065-19.2019.f7-1 Extended Data Figure 7-1 Modulation of β and γ power by the phase of the respiratory cycle. Average time frequency maps centered on the normalized respiratory cycle, in the three-recorded structures (along the vertical axis) and the four experimental categories (along the horizontal axis). On each graph, the red vertical dotted line represents the transition between inspiration and expiration that was set at 0.4, and the white horizontal dotted line represents the transition between β and γ bands. BLA: *n* = 14; mPFC: *n* = 21; and PIR: *n* = 20. Download Figure 7-1, TIF file.

10.1523/ENEURO.0065-19.2019.f8-1 Extended Data Figure 8-1 Beta (upper table) and Gamma (lower table) bands maximum power throughout the respiratory cycle, ANOVA analysis. * : p≤5x10^-2^, ** : p≤5x10^-3^, *** : p≤5x10^-4^. Download Figure 8-1, DOCX file.

In summary, in the PIR beta power is modulated by the phase of the respiratory cycle during freezing and the pattern of this modulation is changed during USV emission. Gamma Power in the three recording sites is strongly modulated by the phase of the respiratory cycle during both freezing and escape, although presenting slightly different patterns. The emission of USV is associated with drastic changes in the time course of this modulation.

## Discussion

The present study assessed for the first time the impact of 22-kHz USV production on brain dynamics in the network involved in fear expression, including the mPFC and the BLA. We report that USV emission modulates oscillatory activities differentially depending on their frequency band. Specifically, it temporally coincides with an increase in delta and gamma power, and a decrease in theta power. In addition, in the PIR, an increase in beta activity is observed. Some of these changes co-occur with USV-induced respiration changes. Indeed, during USV calls, the coupling observed between respiratory frequency and delta oscillatory frequency during silent freezing is lost, and the time course of gamma and beta power within the respiratory cycle is modified. The present data suggest that USV calls could result in a specific gating of information within the fear network, potentially modulating fear memory, as suggested by our observation that the amount of USV emitted during conditioning is a good predictor of the learned fear response at retention.

### 22-kHz USVs are observed during both passive and active fear responses

22-kHz USVs in rats are emitted in aversive situations such as foot-shock delivery and are considered as reflecting a negative affective state (Litvin et al., 2007; Schwarting and Wöhr, 2012; Brudzynski, 2013). We found that USVs are predominantly produced during freezing, which is consistent with the literature (Brudzynski and Ociepa, 1992; Wöhr et al., 2005; Hegoburu et al., 2011; Shionoya et al., 2013; Boulanger Bertolus et al., 2014; Boulanger-Bertolus et al., 2017). However, we also found that some USVs are emitted during escape. Although some examples of 22-kHz USV during locomotion have been reported (Laplagne and Elías Costa, 2016; Boulanger-Bertolus et al., 2017), the characteristics of these USV remain poorly investigated. Here, we show that 22-kHz USV emitted during escape are shorter and louder than those emitted during freezing and exhibit a higher peak frequency.

We also show that although these two types of USV have globally similar effects on brain oscillations, they present different relationships with performance at 48-h retention. Indeed, while there is a positive correlation between the number of USV freezing during conditioning and the amount of freezing at retention, this correlation is not found for USV escape. This suggests that the two subtypes of 22-kHz USV reflect different aspects of fear response. For instance, USV Escape might be more related to the unconditioned response to shock, while USV freezing are generally considered as part of the conditioned fear response (Wöhr and Schwarting, 2008).

### Freezing and escape differentially modulate brain oscillatory activities

We first investigated whether in the absence of USV emission, the way fear is expressed is associated with different changes in brain oscillatory activities. Freezing is a passive defense response while escape is an active response. Recent studies pinpointed that active and passive fear responses involve distinct and mutually inhibitory neurons in the central amygdala (Gozzi et al., 2010; Fadok et al., 2017). Here, we show that these two response modes also differentially modulate oscillatory activity in the fear circuit. Indeed, compared to escape, freezing is characterized by a higher delta power and a lower θ and γ power. Our data in the delta band are in line with the literature as several recent studies showed that freezing temporally coincides with the development of 4-Hz oscillations in prefrontal-amygdala circuits (Dejean et al., 2016; Karalis et al., 2016; Moberly et al., 2018). In awake animals, θ activity is known to occur preferentially during voluntary locomotor activities (Vanderwolf, 1969; Buzsáki, 2002), thus explaining the increase in theta power observed here during escape. Finally, the increase in gamma power observed during escape might reflect an increased emotional level compared to freezing. Indeed, previous studies both in humans and animals have shown that gamma oscillations are enhanced during emotional situations (for review, see Headley and Paré, 2013; Stujenske et al., 2014; Concina et al., 2018).

### 22-kHz USV emission alters respiration and is associated with changes in oscillatory activities

While several studies have investigated the neural circuit involved in USV production (for review, see Schwarting and Wöhr, 2012), and the correlates of USV perception in the brain of conspecifics receivers (Sadananda et al., 2008; Parsana et al., 2012; Roberts and Portfors, 2015), to our knowledge no study has assessed the effect of USV production on the sender animal’s brain oscillatory activities. We showed that USV emission coincides with an increase in delta power and a decrease in θ power. In addition, an increase in gamma power is observed. The effects are globally similar for both types of USV, and in the three recording sites although small differences exist. Furthermore, a strong increase in beta power is more specifically observed in the PIR.

Importantly, some of the changes observed during USV emission co-occurred with changes in respiratory rhythm. USVs are produced during expiration on constriction of the vocal folds resulting in an increase in subglottal pressure and a reduction of airflow through the nose (Riede, 2011; Sirotin et al., 2014). Consequently, 22-kHz USV emission induces drastic changes in the shape and frequency of the respiratory signal (Frysztak and Neafsey, 1991; Hegoburu et al., 2011; Boulanger-Bertolus et al., 2017). It is known from a long time that respiration drives oscillations time-locked to breathing cycles in the olfactory pathways (Adrian, 1942; Fontanini and Bower, 2005; Buonviso et al., 2006; Kay et al., 2009; Courtiol et al., 2011; Esclassan et al., 2012; Zelano et al., 2016) and modulates the amplitude of local beta and gamma oscillations in the olfactory bulb (Buonviso et al., 2003; Cenier et al., 2009; Rosero and Aylwin, 2011) and in the olfactory cortex (Fontanini and Bower, 2005; Zelano et al., 2016). Several recent papers have highlighted that outside its impact on olfactory regions, nasal respiration also entrains oscillations in widespread brain regions in awake rodents (Ito et al., 2014; Nguyen Chi et al., 2016; Biskamp et al., 2017; Zhong et al., 2017; Rojas-Líbano et al., 2018; Tort et al., 2018b) and modulates the amplitude of fast oscillations (Ito et al., 2014; Biskamp et al., 2017; Zhong et al., 2017). Importantly a recent work has specifically investigated the link between respiration and freezing-related 4-Hz oscillation in the mPFC (Moberly et al., 2018), and reported that during freezing, mice respiratory frequency is correlated with the 4-Hz oscillation in the mPFC, and that disruption of olfactory inputs to the mPFC significantly reduces the 4-Hz oscillation.

**Figure 10.**
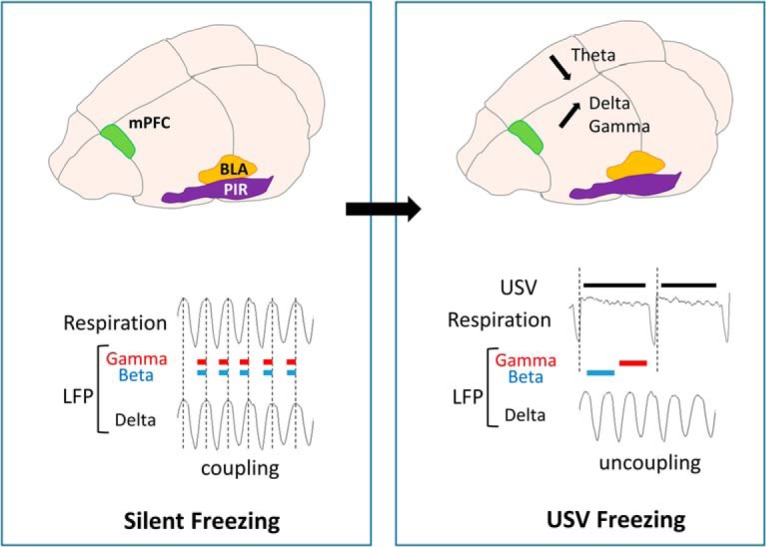
Schematic summary of the data obtained during silent freezing and USV freezing. During silent freezing, delta frequency covaries with nasal respiratory frequency. In addition, power in the beta band for the PIR and in the gamma band for the three recording sites is modulated in phase with respiration, with higher beta and gamma power during inspiration than expiration. USV freezing emission coincides with a decrease in theta power and an increase in delta and gamma power. In parallel, a deep slow-down of respiratory frequency is observed, with the uncoupling between delta frequency and respiratory frequency. Furthermore, a reorganization of beta and gamma activity power during the respiratory cycle occurs, with increased β power in the PIR during the first half of expiration phase, and increased gamma power in the three recording sites during the second half of expiration.

Here, we show that during silent freezing, dominant frequency in the delta band covaries with respiratory frequency. In addition, we report that the activity in the gamma band for the three recording sites, and in the beta band for PIR, are modulated in phase with respiration, with greater beta and gamma power during inspiration than expiration (see summary in Fig. 10). Importantly we also show that USV emission, particularly during freezing, coincides with important changes in the relationship between respiration and oscillatory activity. First, USV emission induces a deep slow-down of respiratory frequency. In parallel, the coupling between δ band dominant frequency and respiratory frequency is lost. In addition, a reorganization of beta and gamma activity power during the respiratory cycle occurs, with increased beta power in PIR during the first half of expiration phase, and increased gamma power in the three recording sites during the second half of expiration.

### Functional interpretation

How can we integrate the present data to the existing literature? Respiration-locked oscillations in non-olfactory regions were shown to depend on nasal airflow (Ito et al., 2014; Yanovsky et al., 2014). Indeed, olfactory sensory neurons have mechanosensitive properties (Grosmaitre et al., 2007) and the signals elicited by rhythmic airflow in the nose are transmitted to the olfactory bulb and the PIR (Fontanini et al., 2003; Wu et al., 2017). The PIR has direct connections with the PFC (Clugnet and Price, 1987) and the olfactory information has unique direct access to the amygdala (McDonald, 1998). We propose that the deep slow-down of respiratory rate added to the reduction of airflow through the nose during USV calls (Riede, 2011; Sirotin et al., 2014) is responsible for the loss of coupling between nasal rhythm and δ oscillation. During USV calls, brain delta oscillations become independent of nasal respiration and their power increases. In parallel, beta and gamma activity power increases during expiration. Interestingly, Manabe and Mori (2013) reported that in olfactory regions, gamma oscillations can lock to different phases of the respiratory cycle depending on the animal’s behavior. Indeed, during active exploration with high sniffing rate, gamma is phase-locked to inhalation, while during awake resting in which rats show a slow respiration rate with long exhalation phase, gamma is phase-locked to exhalation. The authors propose that gamma oscillatory coupling can be generated either by olfactory sensory inputs during inhalation, or centrally in the brain during exhalation. The same kind of interpretation could hold for our data, with the emission of USV being associated with a reorganization of gamma coupling during expiration, potentially resulting in a different gating of information to downstream structures of the fear network. Since we observed that the amount of USV emitted during freezing at training is correlated with the learned freezing response at retention, we suggest that the window of a USV call and its particular respiratory pattern added to its specific combination of brain oscillatory activity, might enhance plasticity at given sites of the network and ultimately strengthen long-term fear memory. Additional experiments are needed to explore the causal link between USV-related changes in oscillatory activities and fear memory.

A better knowledge of the impact of USV production on brain neural dynamics is not only important for understanding the respective weight of the different components of fear response, but is also particularly relevant for rodent models of human neuropsychiatric disorders, for which socio-affective communication is severely impaired (Wöhr and Scattoni, 2013).

## Synthesis

Reviewing Editor: Carmen Sandi, Swiss Federal Institute of Technology Decisions are customarily a result of the Reviewing Editor and the peer reviewers coming together and discussing their recommendations until a consensus is reached. When revisions are invited, a fact-based synthesis statement explaining their decision and outlining what is needed to prepare a revision will be listed below. Note: If this manuscript was transferred from JNeurosci and a decision was made to accept the manuscript without peer review, a brief statement to this effect will instead be what is listed below.

In this manuscript, the authors have successfully incorporated suggestions from two JN reviewers. This novel and important study addresses the link between ultrasonic social vocalizations (USV), specifically 22 kHz USV, (odor) fear conditioning, respiration and activity in task-associated brain areas (BLA, mPFC and olfactory piriform cortex). Using a powerful combination of recording approaches, the study shows that whereas USV calls are paralleled by increases in delta and gamma power and decrease in theta power, during its emission there is no coupling between respiratory rate and delta frequency (which otherwise happens during silent freezing). The activity during olfactory fear acquisition seems to predict freezing behavior during retrieval and thus, memory strength. Based on these findings, the authors put forward a hypothesis whereby “sequences of USV calls could result in a differential gating of information within the network of structures sustaining fear behavior, thus potentially modulating fear expression/memory”. Therefore, this study is an important step forward to understand how USV and respiratory rhythms influence coordinated brain activity during defensive and emotional states.
