# Supplementary figures and images for: New Insights from 22-kHz Ultrasonic Vocalizations to Characterize Fear Responses: Relationship with Respiration and Brain Oscillatory Dynamics
Source: eNeuro. 2019 May 7;6(2):ENEURO.0065-19.2019. doi: 10.1523/ENEURO.0065-19.2019 (PMC6506822; doi:10.1523/ENEURO.0065-19.2019)

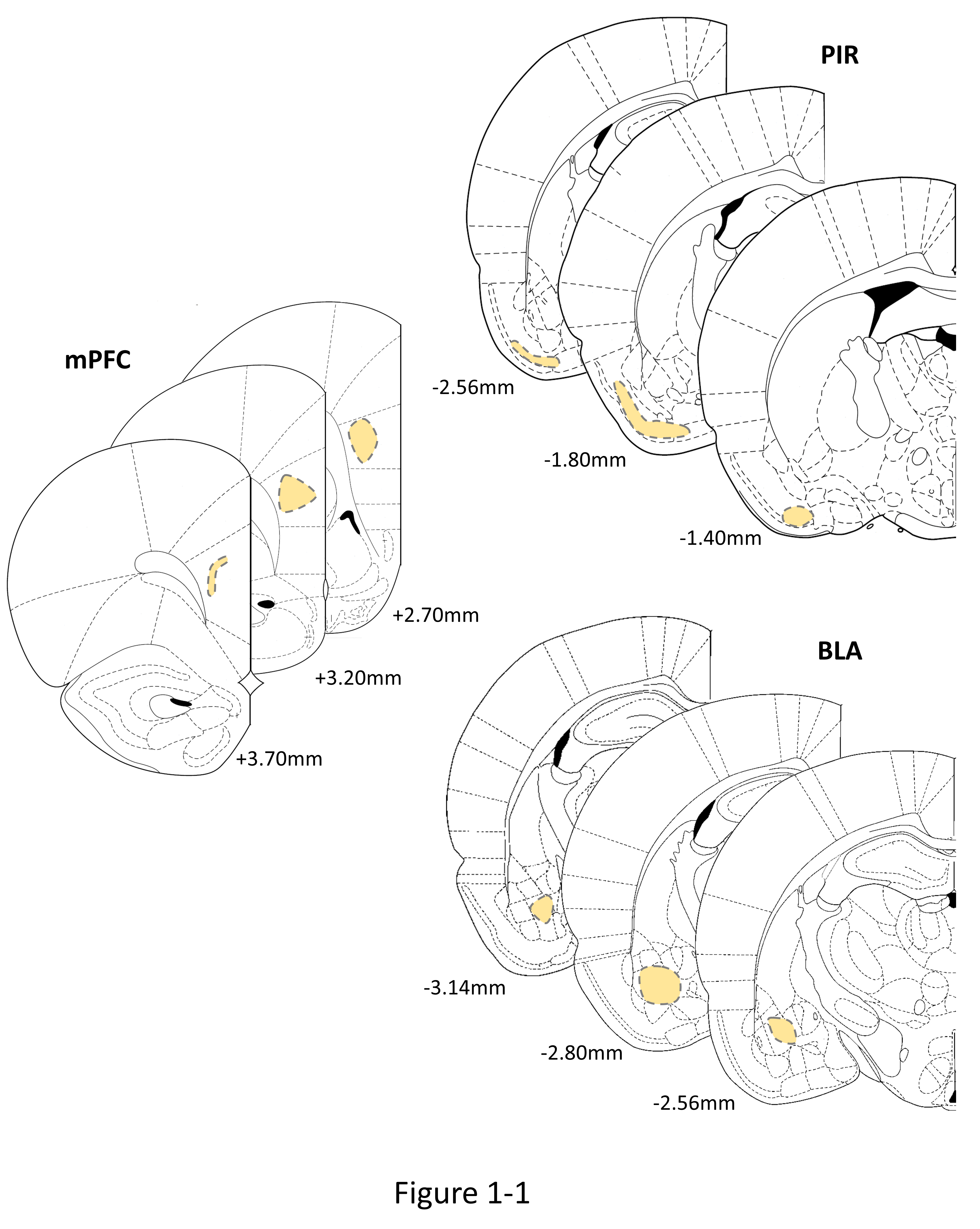

Supplement: Extended Data Figure 1-1 — Areas targeted by the electrodes (light orange areas) in the three recording sites. Numbers at the bottom indicate the relative position of coronal slices from bregma (adapted from Paxinos and Watson, 2007). mPFC: n = 21; PIR: n = 20; and BLA: n = 14. Download Figure 1-1, TIF file. [file sup_enu-eN-NWR-0065-19-s01.tif]

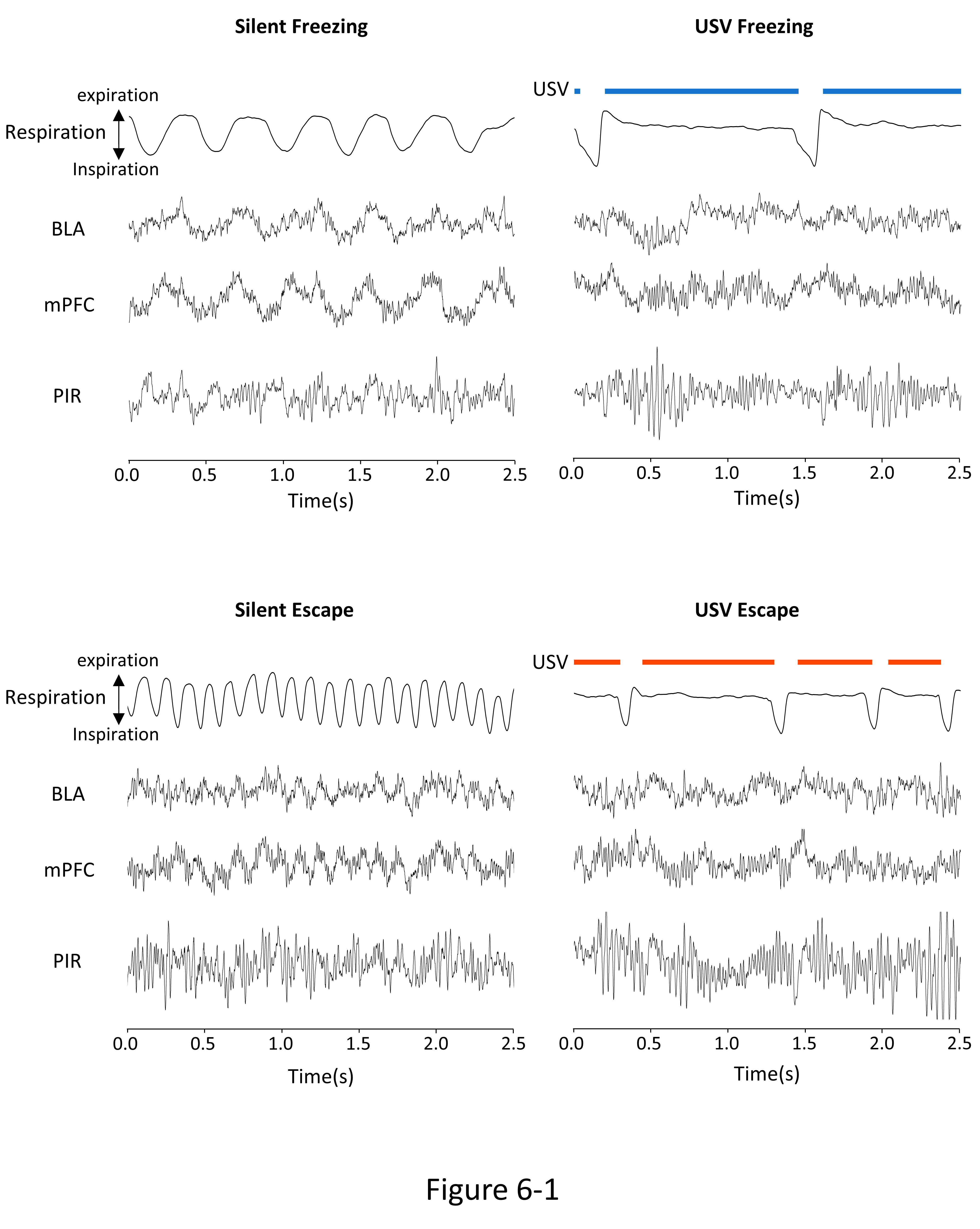

Supplement: Extended Data Figure 6-1 — Examples of raw traces obtained in the same animal in the three recording sites and the four experimental categories. Each panel represents from the top: USVs calls (for the panels on the right), raw respiratory signal, and LFP signals recorded in the BLA, mPFC, and PIR. Download Figure 6-1, TIF file. [file sup_enu-eN-NWR-0065-19-s02.tif]

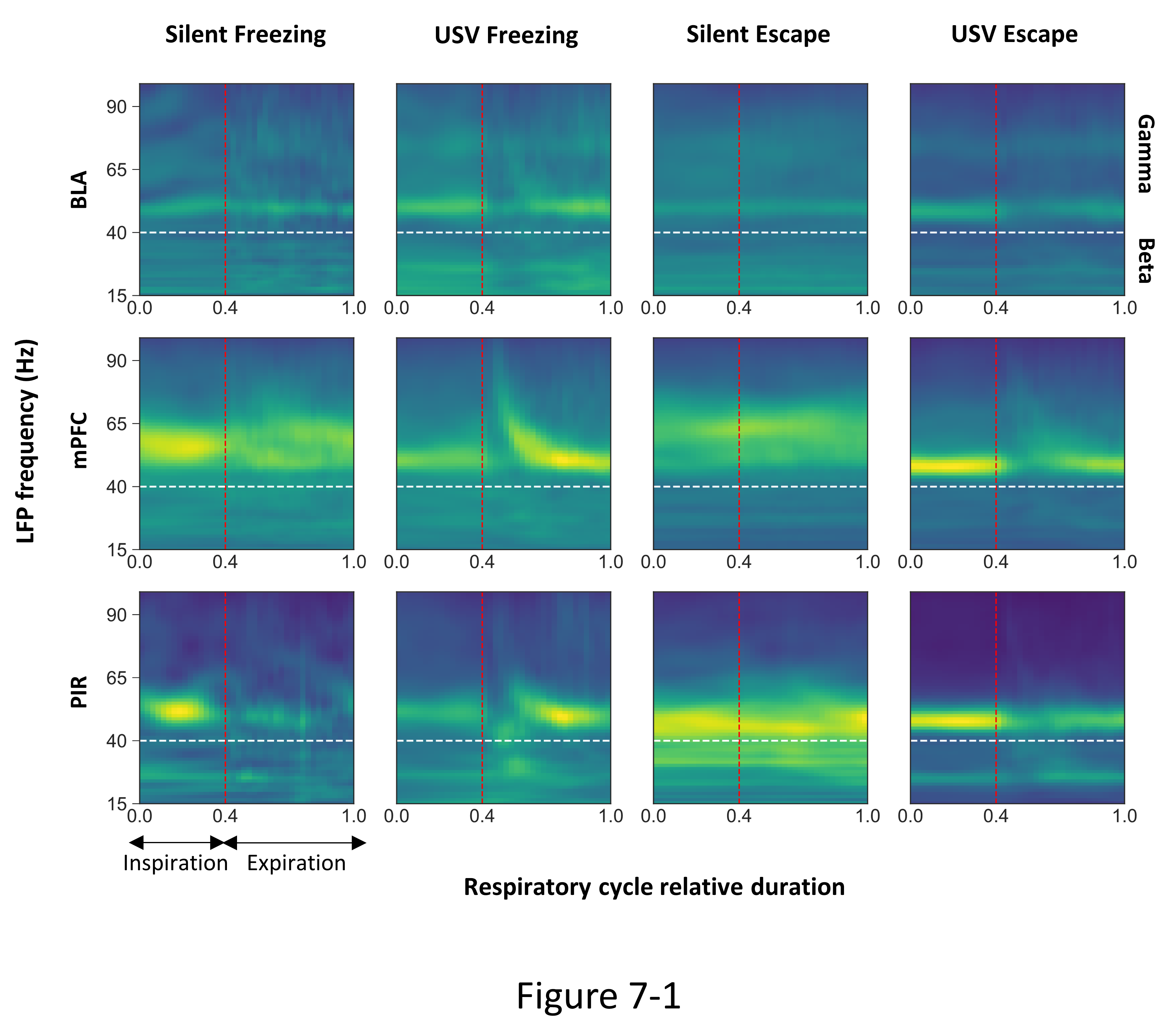

Supplement: Extended Data Figure 7-1 — Modulation of β and γ power by the phase of the respiratory cycle. Average time frequency maps centered on the normalized respiratory cycle, in the three-recorded structures (along the vertical axis) and the four experimental categories (along the horizontal axis). On each graph, the red vertical dotted line represents the transition between inspiration and expiration that was set at 0.4, and the white horizontal dotted line represents the transition between β and γ bands. BLA: n = 14; mPFC: n = 21; and PIR: n = 20. Download Figure 7-1, TIF file. [file sup_enu-eN-NWR-0065-19-s03.tif]
