# Supplementary material for: New Insights from 22-kHz Ultrasonic Vocalizations to Characterize Fear Responses: Relationship with Respiration and Brain Oscillatory Dynamics
Source: eNeuro. 2019 May 7;6(2):ENEURO.0065-19.2019. doi: 10.1523/ENEURO.0065-19.2019 (PMC6506822; doi:10.1523/ENEURO.0065-19.2019)
Supplement: Extended Data Figure 8-1 — Beta (upper table) and Gamma (lower table) bands maximum power throughout the respiratory cycle, ANOVA analysis. * : p≤5x10^-2^, ** : p≤5x10^-3^, *** : p≤5x10^-4^. Download Figure 8-1, DOCX file. [file sup_enu-eN-NWR-0065-19-s07.docx]

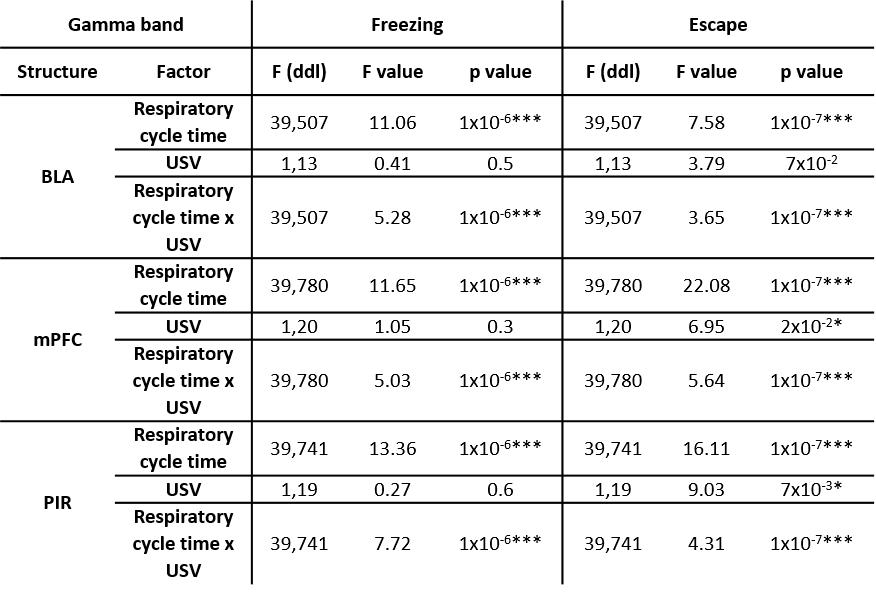

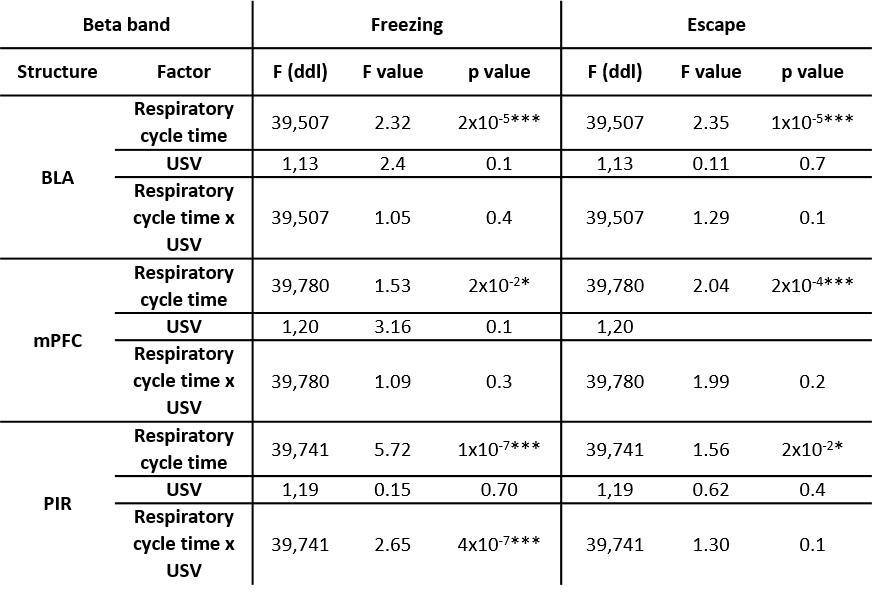


**Figure 8-1:** Beta (upper table) and Gamma (lower table) bands maximum power throughout the respiratory cycle, ANOVA analysis. * : p≤5x10^-2^, ** : p≤5x10^-3^, *** : p≤5x10^-4^.
